# Network Reconstruction Based on Proteomic Data and Prior Knowledge of Protein Connectivity Using Graph Theory

**DOI:** 10.1371/journal.pone.0128411

**Published:** 2015-05-28

**Authors:** Vassilis Stavrakas, Ioannis N. Melas, Theodore Sakellaropoulos, Leonidas G. Alexopoulos

**Affiliations:** 1 Department of Mechanical Engineering, National Technical University of Athens, Heroon Polytechniou 9, Zografou 15780, Greece; 2 European Bioinformatics Institute (EMBL-EBI), Hinxton, Cambridge CB10 1SD, United Kingdom; Institute Biomedical Research August Pi Sunyer (IDIBAPS)—Hospital Clinic of Barcelona, SPAIN

## Abstract

Modeling of signal transduction pathways is instrumental for understanding cells’ function. People have been tackling modeling of signaling pathways in order to accurately represent the signaling events inside cells’ biochemical microenvironment in a way meaningful for scientists in a biological field. In this article, we propose a method to interrogate such pathways in order to produce cell-specific signaling models. We integrate available prior knowledge of protein connectivity, in a form of a Prior Knowledge Network (PKN) with phosphoproteomic data to construct predictive models of the protein connectivity of the interrogated cell type. Several computational methodologies focusing on pathways’ logic modeling using optimization formulations or machine learning algorithms have been published on this front over the past few years. Here, we introduce a light and fast approach that uses a breadth-first traversal of the graph to identify the shortest pathways and score proteins in the PKN, fitting the dependencies extracted from the experimental design. The pathways are then combined through a heuristic formulation to produce a final topology handling inconsistencies between the PKN and the experimental scenarios. Our results show that the algorithm we developed is efficient and accurate for the construction of medium and large scale signaling networks. We demonstrate the applicability of the proposed approach by interrogating a manually curated interaction graph model of EGF/TNFA stimulation against made up experimental data. To avoid the possibility of erroneous predictions, we performed a cross-validation analysis. Finally, we validate that the introduced approach generates predictive topologies, comparable to the ILP formulation. Overall, an efficient approach based on graph theory is presented herein to interrogate protein–protein interaction networks and to provide meaningful biological insights.

## Introduction

Signaling pathways are of the utmost importance for understanding cellular function and predicting response to environmental perturbations [1–7]. Extensive collections of signaling pathways have been made available to online databases, obtained either from dedicated experiments, computational predictions or obtained manually from research articles. However, most of these interactions lack biological context (cell type, treatments etc.). Thus, even with all these resources available, compiling a context specific network is a tedious and challenging task [8]. On this front computational methodologies have been proposed that combine prior knowledge of protein interactions with experimental data in an attempt to uncover signaling pathways that appear to be functional in the interrogated cell/tissue type.

Most of the computational methodologies for reconstructing signaling pathways based on proteomic data, first employ a modeling methodology to describe formally how signal propagates from one protein to the next in the pathway, and then employ a parameter estimation approach to identify optimal values of the model parameters, in an attempt to make the model best fit the measured data. Common approaches for modeling signal transduction include modeling via Ordinary Differential Equations (ODEs) [9–11], probabilistic/bayesian methods [12], and the various forms of logic modeling such as Boolean and constrained fuzzy logic [13–18]. Deciding on the optimal modeling formalism is not trivial and depends on the prior knowledge available in the literature, the experimental data available for training the model, and the scope of the analysis. For example, ODEs are best used for the quantitative modeling of small scale pathways, when there is available data for all signaling molecules and the protein connectivities are known with a great degree of confidence. Then the pathway reconstruction problem is formulated as the optimal identification of the kinetic constants to minimize the deviation of model predictions and experimental measurements. On the other hand, logic modeling is best used in medium to large scale networks, when there is great ambiguity on the protein connectivities, and data is available for only a subset of the included proteins. Then the pathway reconstruction problem is formulated as the identification of optimal subsets of the prior knowledge network, conserving in the solution only the reactions that appear to be functional based on the data at hand.

Depending on the modeling formalism used, different parameter estimation methods are best employed for identifying the model parameters. Typically, ODE modeling is best coupled with sensitivity analysis methods, while logic modeling is best coupled with optimization methods. In sensitivity analysis, first bibliographic values for the kinetic constants are used, and then the model is simulated under small variations of these parameters providing an estimate of the parameter values that best reproduce the experimental data [19]. A form of “top-down” sensitivity analysis to quantify the input-output relations and molecular interactions in regulatory networks, is presented in [20], where the control of the input signal over the output target is quantified as the ratio of the input-to-output changes at steady state. Such a top-down analysis can be applied to any cellular network despite its complexity. In optimization based methods an objective function is introduced representing the deviation of model predictions from the experimental data, and formal algorithms such as particle swarm optimization, Linear Programming formulations, and Genetic Algorithm are used to identify optimal values of model parameters, minimizing the objective function and thus, best fitting the data at hand.

These mechanistic methodologies, can be implemented without the incorporation of experimental data. Nevertheless, their predictive efficiency is limited due to constraints imposed by the accuracy of the proteins’s connectivity in the pathways used as a scaffold [21, 22]. As a result, if the initial topology, as adopted from literature, represents the protein’s connectivity inaccurately, then the extracted topology will yield significant error in the final model. To avoid this particularity, experimental data are usually combined with a training algorithm, to calibrate the model to best fit the experimental design [23–25].

The approach described herein, is based on graph theory, which serves as a powerful mathematical modeling tool for the analysis of biochemical networks. Such networks consist of entities that represent several type of biomolecules, as proteins and genes [26–32]. Over the past decade, several computational approaches have been published investigating how extracellular signals, propagated through cell’s biochemical microenvironment, regulate cellular responses [33]. Some of them utilize Floyd-Warshall algorithm [34] to compute shortest paths between network’s components, with the aim of representing the underlying molecular mechanisms of genetic interactions [28]. Other approaches, such as the SST algorithm described in [29], focus on calculating Spanning Trees to model significant quantities in biochemical networks. While these approaches address different problems, ours addresses the network reconstruction based on proteomic data and prior knowledge of protein connectivity. However, both these approaches and ours use the same formalism to model signal transduction. Additional graph theory tools can further enhance computational approaches on pathway construction.

Our approach constitutes a novel methodology for modeling medium and large scale signaling pathways based on experimental data and is meant to serve as an alternative to the logic modeling-optimization pipeline. More specifically, we start with a set of dependencies between signaling molecules as extracted from the experimental data and then using a breadth-first transversal of the graph, we compute the shortest paths of the prior knowledge network that fit these dependencies. Moreover, using several algorithmic approaches, we are able to handle conflicts in the dependencies to extract the most meaningful (biologically) pathways.

Instead of an optimization algorithm or a sensitivity analysis approach, using a breadth-first transversal approach does not force the user to decide on a mathematical formalism, since most of the pathways are obtained in a graph form. Thus, adopting a mathematical formalism is a logic leap. Furthermore, since optimization algorithms are typically NP-hard problems, to impose the experimental dependencies, we take advantage of the breadth-first transversal complexity of O(*V* + *E*), let V stand for the total number of the vertices in the graph and E for the total number of the edges. As a downside, we cannot guarantee global optimum, but using heuristic algorithms, we are able to identify the minimum supersets of the graph, where the optimum solution lies in.

## Materials and Methods

This section presents the methodology used to develop the algorithm based on the fundamentals of graph theory. First, we describe the basic principles, on top of which, we build our signaling topologies. Next, we analyse the reasoning we employ to set the dependencies of our problem, as extracted from the experimental design. Finally, we illustrate the major algorithmic steps of our formulation via a toy model demonstration, as an oversimplification of realistic signaling scenarios.

### Basic Definitions

Graphs are mathematical structures consisted of entities (formally called vertices and less formally nodes or compounds) and relations between them (formally termed edges). When it comes to modeling of biochemical networks, these entities typically represent proteins, genes or other type of biomolecules [27, 35]. In our case, we focus on proteomic analysis and we assume that we are given a directed graph G = (V, E) capturing our prior knowledge on the signaling topology and a set of experimental scenarios coupled with a set of measurements. The graph nodes constitute the variables of our formulation, indicating the state of a protein. A protein can be in one of the two states: state 0, where the protein is inactive and can’t transduce signal and state 1 where the protein is active/phosphorylated and can transduce signal downstream. These nodes are indexed by i ∈ *I*_*V*_, *I*_*V*_ = {1, …, *n*_*V*_}. The graph edges constitute the network reactions indexed by j ∈ *I*_*E*_, *I*_*E*_ = {1, …, *n*_*E*_}. Each reaction is associated with a reactant *R*_*j*_ and a product *P*_*j*_. A pathway is defined as a set of reactions *I*_*R*_ ⊆ *I*_*E*_ and species *I*_*v*_ ⊆ *I*_*V*_. While typically the set of species, included in the signal process, is well known from the experimental scenarios, the set of reactions in the final network is uncertain. Thus, the goal of the proposed formulation is to find a subset of reactions and nodes out of all the possible candidates, that best describes the data at hand. We perceive the initial topology as an edge list, e.g., *R*_*j*_ → *P*_*j*_.

In our framework, an experiment is defined as a set of experimental scenarios coupled with a set of measurements for each scenario and is represented as a data matrix, where each row (r) correspond to a stimulus used to perturb the cells and each column (c) to a measured signal. Each matrix element *x*_*rc*_ represents a Boolean variable signifying the measured state of the signal c upon stimulation with molecule r, where 0 indicates unchanged phosphorylation level and 1 activated phosphorylation level. Note that the proposed approach manages a qualitative description of the experiments and handles single treatment data (no stimuli combination). In Table 1 we present an example of an experimental data matrix, to indicate how we set the experimental dependencies of our problem.

**Table 1:** Data matrix of 2 considered experimental scenarios.

	RAF-1	ERK	AP1	GSK-3	P38	NFKB	IKK	MAP3K1	MAP3K7	PI3K
EGF	1	1	1	1	1	1	1	1	1	1
TNFA	0	0	1	1	1	1	0	0	0	0

Herein, we present fictitious signaling events for 10 signals downstream of EGF and TNFA stimulation. Each row corresponds to one experimental scenario and each column contains the measured state changes of the readout species. If a node is regulated in the respective scenario, then *x*_*rc*_ = 1, otherwise *x*_*rc*_ = 0. For instance, we imply that TNFA stimulus causes an activation of AP1 signal (*x*_23_ = 1) and a zero response of IKK measured species (*x*_27_ = 0).

For every phosphorylation event observed (*x*_*rc*_ = 1) in the data matrix, we infer a causal relationship or dependency between *r*_*th*_ signaling molecule and *c*_*th*_ signal. All the dependencies are collected, as a list of subordinate relationships between a source node (stimulus) and a target node (signal) and are indexed by k. The symbol → implies a desired intermediate pathway. According to Table 1, we set the fourteen dependencies presented in Table 2.

**Table 2:** Setting the dependencies.

k	Dependencies
1	EGF → RAF-1
2	EGF → ERK
3	EGF → AP1
.	.
.	.
.	.
14	TNFA → NFKB

We present the 14 dependencies extracted from the experimental matrix presented in Table 1. The symbol → signifies the desired intermediate pathway between the two molecules.

The goal of the proposed methodology is to infer a network that best describes the dependencies indicated by the experimental data. An intuitive approach would be to connect each stimulus used to perturb the cells, with the activated signals, through the shortest path possible. If such a path exists and it doesn’t include a node that has been measured as inactive, i.e. it doesn’t contradict the data, then we imply that we have a Direct Path from the stimulus to the measured signals. However, if there is no shortest path, free of conflict nodes, connecting the stimulus to the active signal, then we employ an heuristic process to handle these cases and come up with alternative paths.

### Algorithmic procedure

**Fig 1:**
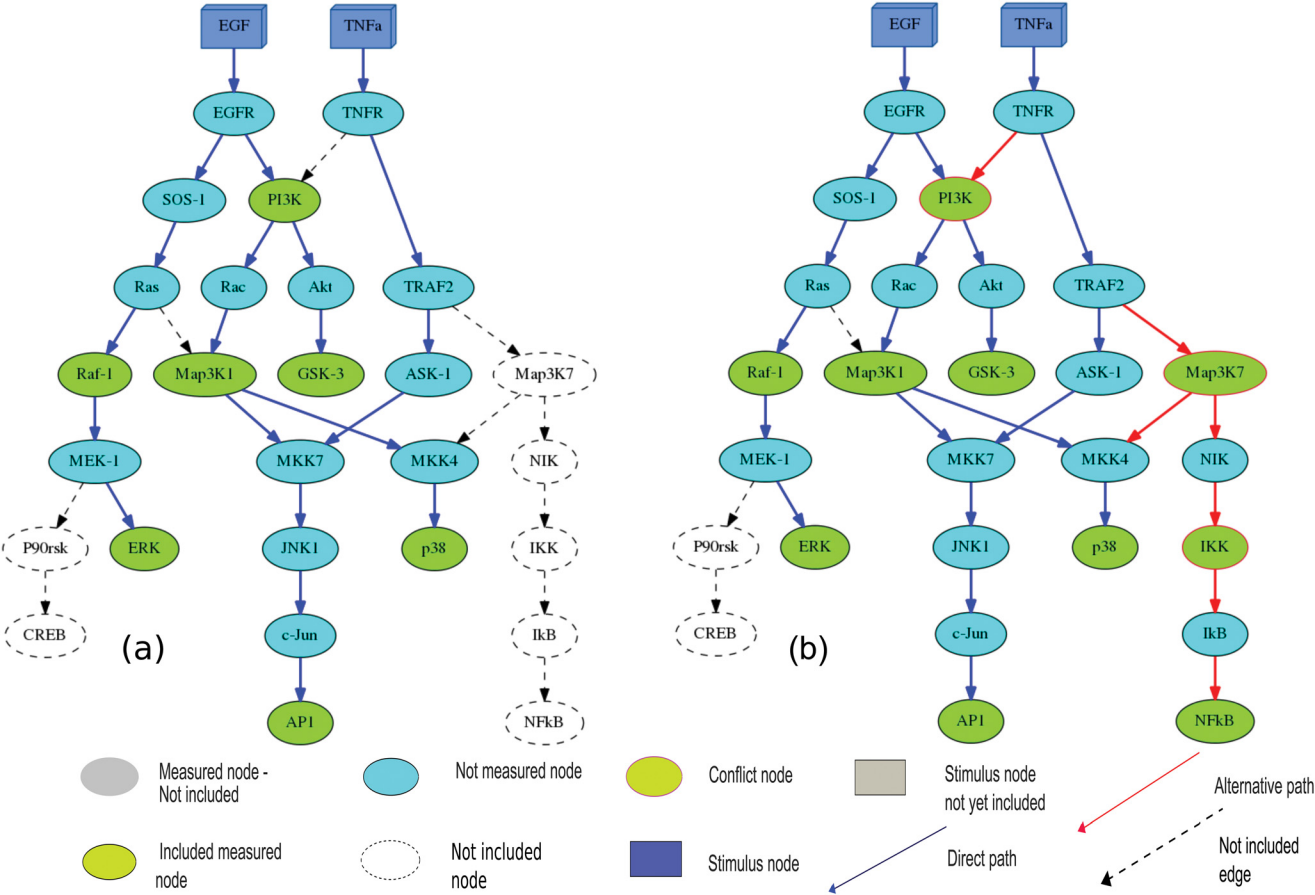
A simple example network used for illustration purposes—Workflow. (a) The full network adopted from [36], after applying the Direct Paths step. These Direct Paths are depicted in blue edges, while in dashed we present edges and nodes not yet included in our solution. (b) The compressed model, as obtained after applying the Alternative Paths step and dealing with conflicts detected in the network. In this compressed version of the network we notice the appearance of the connection between TNFR and PI3K. The purpose of this new edge is not to link TNFA to P38 phosphorylation, but to satisfy the TNFA → GSK-3 dependency. The fact that TNFA links to P38 phosphorylation through this connection (i.e. TNFR → PI3K) is coincidental in this case and depends on the paths derived via the “Direct Paths” procedure (the blue edges have already been included in the final topology from the previous step). The algorithm, in order to satisfy the TNFA → P38 dependency chooses the shortest path TNFA → TNFR → TRAF2 → MAP3K7 → MKK4 → P38, including two conflicts (i.e. MAP3K7 and IKK nodes have been measured as inactive under TNFA stimulation). However, this error vanishes due to satisfaction of the two dependencies (TNFA → P38 and TNFA → NFKB). Consequently, the scoring method assesses this case as a draw case (2 Satisfied Dependencies – 2 Conflicts Detected = 0). In this work we suggest that the draw cases should be included in the compressed topology, as they add connectivity-topology information. The algorithmic steps and the experimental design is colour annotated. In blue we present the Direct Paths produced in the previous step, while in red we present the Alternative ones. The nodes in crimson contours represent the detected inconsistencies (conflicts) between network topology and experimental measurements. Finally, in dashed we present edges and components excluded from the final solution.

Having introduced the fundamental concepts of our approach, we proceed to describe the major algorithmic steps, using in parallel an imaginary, but biologically plausible example, as presented in Fig 1(a) and 1(b). The PKN includes a subset of intracellular signaling networks known to be activated downstream of EGF and TNF stimulation, and was derived from a larger network presented in [36]. For this PKN, we developed a fictitious data of 2 stimuli and 10 signals, presented in Table 1. The readouts chosen (depicted in light green color) are established downstream events of EGF/TNF stimulation (depicted in blue color). The PKN is a Directed Acyclic Graph (DAG). Although our method is not limited to that structure.

The first step of our method is to identify the Direct Paths that satisfy the imposed dependencies. To do so, we employ the Floyd-Warshall algorithm, to compute the shortest paths in the PKN. Floyd-Warshall algorithm is a classic graph analysis algorithm for computing shortest paths in a weighted graph with positive or negative edge weights (but with no negative cycles) and solves the all-pairs shortest-path problem in O(*V* ^3^) steps [34], [37]. By executing the algorithm, we find the lengths (summed weights) of the shortest paths between all pairs of vertices and we store them in a *n*_*V*_ × *n*_*V*_ matrix. Since we do not use some probabilistic approach, we attach to each interaction weight equal to +1.

In our toy model, we start with a list of fourteen dependencies, as presented in Table 2. We notice that, all activation events downstream EGF are equal to 1, so ideally we have to connect EGF with all the measured signals. However, such an attempt is not possible due to limitations dictated by the generic topology (i.e., MAP3K7, IKK and NFKB signals are not reachable). Fig 1(a) visualizes the Direct Paths hypothesis. It is apparent, that via Direct Paths, we can satisfy the remaining EGF dependencies. On the other hand, under TNFA treatment all the activation events are reachable based on the topology. However, only AP1 is reachable through a Direct Path, because of inconsistencies detected in the remaining dependencies. To satisfy the remaining dependencies, it is essential to handle these conflicts in the respective Direct Paths, through Alternative Paths.

To identify Alternative Paths, satisfying the remaining dependencies, we developed a custom searching method, based on the Breadth-First-Search algorithm. The main idea is to find alternative starting points through BFS searching method and then to use Floyd-Warshall matrix to find conflict free paths. We begin from the stimuli level, exploring all the neighboring nodes. Then, for each of those nearest nodes, we explore their unexplored neighbor nodes, and so on, until we reach the measured signal. At each iteration of BFS, we check the Floyd-Warshall matrix for conflict free pathways, accessing new shortest paths from the new starting points towards the measured signals. If such pathways are not identified, we implement a pathway scoring method to satisfy the remaining dependencies.

Our scoring method is a systematic way to check and score the paths that can satisfy the unmet dependencies. For each unmet dependency and for every potential pathway satisfying this dependency (starting from the shortest and continuing to the Alternative Paths produced in the previous steps) we check whether the number of dependencies served by a specific pathway is greater or equal to the number of the conflict nodes introduced to the network. If this is the case, the path is included in the compressed model and the searching continues to the next unmet dependency.

Finally, if there is still a list of unmet dependencies, we continue applying the same scoring methodology, only this time we nullify the detected conflict nodes, which have already been included in the pathway reconstruction of previous steps. The main idea underlying this intuition is that some conflict nodes may not facilitate the satisfaction of a single dependency, but a combination of them, contributing in the cumulative satisfaction of multiple dependencies.

Fig 1(b) visualizes the final compressed network model after the Alternative Paths and scoring method implementation. In this final solution, we notice that extra TNFA dependencies (TNFA → P38, TNFA → NFKB and TNFA → GSK-3) are satisfied. Considering the experimental scenarios (Table 1), there is no activation of MAP3K7, IKK and PI3K signals downstream TNFA stimulation. However, including MAP3K7 signal in the final model, allows the satisfaction of the TNFA → P38 dependency. Implementing our scoring method in the specific path: 1 Dependency (TNFA → P38)—1 Conflict detected (MAP3K7) = 0. Same applies to the case of TNFA → NFKB dependency: 2 Dependencies (TNFA → NFKB, TNFA → P38)—2 Conflicts detected (MAP3K7, IKK) = 0 and to the case of TNFA → GSK-3 dependency (PI3K only intermediate conflict node detected). The alternative edge TNFR → PI3K creates also additional pathways, satisfying already met dependencies (TNFA → P38 and TNFA → AP1). It enriches the final model with information about the protein connectivity, without affecting the total goodness of fit, as the error included (TNFA observes MAP3K1, which is not accurate based on the Table 1) vanishes meeting the dependencies EGF → P38, EGF → AP1, EGF → MaP3K1 and TNFA → GSK-3.

Having identified the subset of reactions comprising the derived model, we access the quality of our solution. To do so, we calculate the goodness of fit, as a metric of the quality of our approach. We compute a compressed model matrix, where each row corresponds to a stimulus used to perturb the cells and each column to a measured signal. Apparently, this new matrix and the experimental data matrix have the same size. If the *c*_*th*_ measured node is reachable under stimulation of *r*_*th*_ perturbed node, based on the compressed model’s topology, we set the corresponding matrix element $x_{rc}^{cm}$ = 1, otherwise we set $x_{rc}^{cm}$ = 0. For instance, according to the compressed model of our toy model, presented in Fig 1(b), we set the reachable matrix presented in Table 3.

**Table 3:** Reachable signals matrix according to the compressed model’s topology.

	RAF-1	ERK	AP1	GSK-3	P38	NFKB	IKK	MAP3K1	MAP3K7	PI3K
EGF	1	1	1	1	1	0	0	1	0	1
TNFA	0	0	1	1	1	1	1	1	1	1

Herein we present the reachable signals matrix for our toy model, according to the compressed model’s topology. We set a matrix of same size as the experimental matrix presented in Table 1. In this new matrix, each row corresponds to a stimulus used to perturb the cells and each column to a measured signal. If the *c*_*th*_ measured node is reachable under stimulation of *r*_*th*_ perturbed node, based on the compressed model’s topology, we set the corresponding matrix element $x_{rc}^{cm}$ = 1, otherwise we set $x_{rc}^{cm}$ = 0.

Let *n*_*st*_ be the total number of stimuli, *n*_*sig*_ the total number of the signals and *n*_*s*_ the total number of the experimental measurements, we calculate the percentage error as:

$$\begin{array}{c} Error=\sum_{c=1}^{n_{sig}}\sum_{r=1}^{n_{st}}\left|x_{rc}^{cm}-x_{rc}\right|/n_{s}\cdot 100\% \end{array}$$ (1)

### Implementation

We aim to develop a readable and maintainable code that can serve as an entry point into computational biology. All programming procedures were written in the MatLab environment, therefore run in all three most applicable operating systems: GNU/Linux, Microsoft Windows and Apple Os X. The developed algorithm is very easy to use; the user has to provide four files to define network training problem: (i) the network topology in a.txt format using a tab perimeter between the two components of each reaction, (ii) a.txt file containing the stimuli of the experiment, (iii) a.txt file containing the signals of the experiment, and (iv) a.txt file containing the experimentally measured state changes for each scenario. The user may then run the implemented code, as described herein, and a.dot file is produced for the visualization of the final solution. Graph visualization, which is a way of representing structural information as diagrams of abstract graphs and networks, holds a great impact in our approach. Thus, the analysis results are communicated using the open source graph visualization software Graphviz (http://www.graphviz.org/), as an overview graph allows users to quickly visualize hypotheses and shows how they are related to each other. MatLab source code for this method, along with a detailed manual and examples of use, is available at http://ntuabiolab.wikispaces.com/Software.

## Results

### Medium scale network

In order to demonstrate the performance of the proposed approach in a realistic situation, we apply it to a published network topology [38], aiming to identify particularities of the signaling pathways. Network reconstruction was based on signaling reactions reported in literature and databases. The experimental scenarios consist of 5 stimuli and 16 measured key phosphoproteins, as described properly in [4] and presented in Table 4 in discretized format. The initial topology is created around these stimuli and measured phosphoproteins and comprises of 139 reactions. The proposed formulation requires a qualitative view of signal transduction, supporting only two discrete states indicating the variation of the activation state of signaling nodes (“1” for activation and “0” for unchanged state). Thus, the raw data had to be discretized first. Several interesting signaling features can be observed simply by inspection of the discretized experimental dataset.

**Table 4:** Medium scale network experimental data.

	hspb1	akt1	p70s6k	shp2	jnk2	ikba	gsk3b	p38mapk	nfkb	mp2k6	tor	mek1	erk1	rsk1	creb1	rs6
il6	0	0	0	0	0	1	0	0	1	0	0	0	0	0	0	0
tnfa	0	0	0	1	0	0	0	0	1	0	0	0	0	0	0	0
il1b	1	0	0	0	0	1	0	1	1	0	0	1	0	0	1	0
tgfa	0	1	1	1	0	0	1	1	1	1	0	1	1	1	1	1
ins	0	0	0	0	0	0	0	0	0	0	0	0	0	0	0	0

The experimental scenarios, presented here in discretized format, consist of 5 stimuli and 16 measured key phosphoproteins and is described properly in [4]. The proposed formulation requires a qualitative view of signal transduction, supporting only two discrete states indicating the variation of the activation state of signaling nodes (”1” for activation and ”0” for unchanged state.

First, the pro-growth stimuli, Tumor Growth Factor alpha (TGFA) and the inflammatory ligand IL1B cause several activations, including both stimulation factors implying a P38MAPK, NFKB and CREB1 activation. On the other hand, both Tumor Necrosis Factor alpha (TNFA) and Interleukin 6 (IL6) activate only NFKB signal. Additionally, it is likely that PKNs often lack interactions that are supported by data. Indeed, we observe this particularity in the case of the TNFA → SHP2 causal dependency, a pathway not supported by the initial topology. As stated above (see “Basic Definitions” in “Materials and Methods” section), according to the experimental data matrix, we reformulate our data to a list of 22 dependencies.

**Fig 2:**
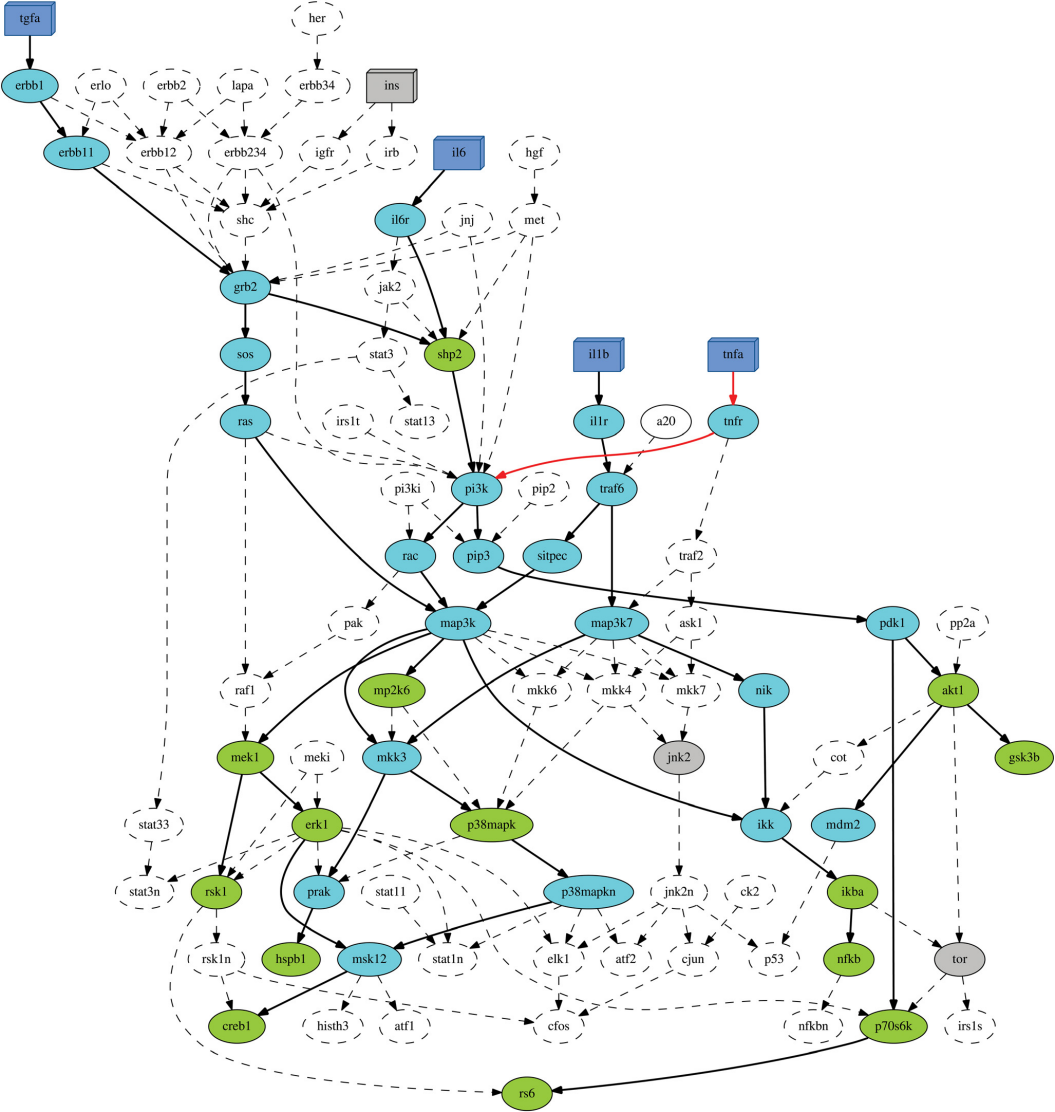
Medium scale network-Compressed model. The model structure can be compressed substantially from 90 nodes and 139 edges to 41 nodes and 44 edges. The compressed model reflects the essential dependencies in the original network structure that can be addressed by the given set of measured nodes. Our solution resulted in a fitting error of 29, which has thus reduced much in comparison to 59 in original model. Several edges are absent due to conflict with the data. One example is the absence of RSK1 → RS6, in order to isolate the RS6 activity from the IL1B stimuli. In a similar manner, several edges are preserved as MEK1 → ERK1 and MEK1 → RSK1 to permit the activity of ERK1 and RSK1 under the TGFA treatment. Additionally, MAP3K → IKK enables the activation of NFKB signal under both IL6 and TGFA stimulation and the activation of IKBA measured node from the IL6 stimulus. In red color, we present the removed edges in the compressed model after a parameter change in our ranking method. This new model structure consists of 38 nodes and 41 edges. The new compressed model reflects essentially the experimental dependencies in the original network structure and provides a final fitting error of 19, much reduced in comparison to 59 in original model and 29 in the previous solution.

We propose changes in the network structure to improve the agreement between experimental data and PKN. Fig 2 shows the compressed topology as derived through the computational procedure. The experimental design is represented by color coding: in blue color we represent the stimulation factors and in green color the measured signals, while the rest of the intracellular proteins are represented in cyan color. Whereas the Insulin (INS) pathway has been included because of its major role in liver homeostasis, the experimental data indicate unchanged states for all the measured key phosphoproteins under conditions of INS stimulation. In addition, signals TOR and JNK2 remain unaffected in all given scenarios. These particularities are demonstrated in grey signs. In dashed contours and lines are visualized no reachable compounds, nodes and edges excluded from the final solution.

Our results describe accurately the signaling pathways downstream major players for liver homeostasis and meet the dependencies imposed by the experimental scenarios. More specifically, we identified several causal relations well known from the literature via the Direct and Alternative paths procedures. For instance, we validated all dependencies under IL1b stimulation and all but one under TGFA treatment. The paths MAP3K → MEK1 → ERK1 → MSK12 and MAP3K → MKK3 → P38MAPK → P38MAPKN → MSK12 constitute two alternative routes (both present in the final solution) to reach the measured signal CREB1. Additionally, with the edge MAP3K → IKK, the model structure comprises an activation route from IL6/TGFA stimuli to IKBA/NFKB signals. We also observe two alternative routes (SOS → RAS and SHP2 → PI3K → RAC—both present in the final solution) to reach MAP3K node starting from GRB2 node.

The current approach aims to adapt the network structure to the data in a automatic way in order to resolve the discrepancies between model and data. Our algorithm was able to detect such discrepancies in the original topology, such as the measured node SHP2 in the experimental dependencies IL6 → IKBA and IL6 → NFKB and IKBA in the dependencies TNFA → NFKB and TGFA → NFKB. To handle these mismatches, our model applies a ranking method for the remaining dependencies, as detailed in Methods. For example, for the case of the SHP2 mismatch in the IL6 → IKBA/NFKB pathways: 2 dependencies (IL6 → IKBA, IL6 → NFKB) served by introducing 1 conflict (SHP2) and for the case of the IKBA mismatch in the TNFA → NFKB pathway: 1 dependency (TNFA → NFKB) served by introducing 1 conflict (IKBA). The latter demonstrates that the draw cases are favoured by our approach, since there is limited evidence to support these causal links. In any case, dedicated experiments are required to support or prove these suggestions.

Training against data generally wields a model having a substantially better fit than the PKN, which is also the case with our data and model. The untrained ensemble containing all possible interactions exhibited a poor fit (59% across the dataset), whereas our trained model halved the initial error (29%), in a total time of 5 seconds. Exploring possible explanations and further estimations for a better fit of our trained model, we inspect Table 4 and we observe that Il6, TNFA and INS stimulations demonstrate significant mismatches with the data, according to the PKN. We asked how we could improve the correlation of cellular responses to phosphoprotein activity. To achieve that, we attempt a parameter change in our ranking method and we exclude the solutions acquired due to the draw cases.

A view of this new solution is presented in Fig 2 with the removal of the red edges. We notice that this attempt excludes the TNFA → NFKB pathway from the new final solution as a draw case (IKBA intermediate conflict node). The trained ensemble demonstrated a final agreement of 19% (in ∼ 5 seconds) with the measured data. Note that all calculations were done on a PC with a 2.13 GHz Intel double core Pentium P6200 CPU (only a single core was used) and 2 GB 1333 MHz DDR3 Memory.

To summarize, through our computational framework, we validated essential biological patterns of the network structure, indicating important aspects of the signaling pathways in hepatocytes. For instance: (1) STAT3 is not activated by TGFA; (2) Phosphorylation of the autocatalytic domain of P70S6 (termed P70S6K in the model) is independent of ERK1; (3) The activation of CREB1 in response to TGFA is likely to be caused by a MEK1 independent route; (4) Phosphorylation of AKT1 is regulated to PI3K activity; and (5) The activation of MEK1 and ERK1 is dependent to MAP3K activation. These results, generated in a automated way, confirm several conjectures formulated in [4]. In addition, by identifying parallel activation routes that cannot be distinguished with the experimental data at hand, we contribute to increase the robustness of the network’s topology and we suggest further experiments to uncover the true wiring diagram of the signaling pathways in the considered cell type.

All the connections in the solution are biologically relevant as they have been mined from literature. However to validate the functionality of the connections in this specific context, follow up experiments are required where key signaling proteins are blocked and the activation of downstream proteins is monitored to deconvolute their connectivity in the network, in similar fashion to the validation study performed in [39]. In this paper, the experimental validation is beyond our scope as we do not address a specific biological problem, and we only score the performance of our algorithm based on its fitness error and the similarity of the constucted topology with that of the ILP.

In the “Supporting Information” section we provide additional arguments to support the interpretation of the medium scale network discussed in this section. To evaluate the sensitivity of our model, we performed a cross-validation analysis, to assess how the results of our method generalize to independent data sets. We tested our algorithm using 500 randomly generated datasets emulating actual experimental data. The number of activations in these matrices was kept constant and equal to the original matrix presented in Table 4. The results are demonstrated in S1 Fig, in S2 Fig and in S3 Fig. This analysis provided evidence that the developed approach produces compressed topologies that are directly dependent on the data at hand. For more information see S1 Text in the “Supporting Information” section.

Next, we evaluate the performance of our methodology in reconstructing large topologies based on phosphoproteomic data. Large scale networks represent cellular function from a systems perspective and integrate in full detail all signaling events that determine cellular response [40]. They typically exhibit increased difficulty compared to the medium scale networks, with regards to the processing time and the required amount of data [41]. As a case study, we validated players and their corresponding pathways in primary human bronchial epithelial cells, by interrogating the signal transduction downstream of 25 stimuli of interest and constructing a compressed model for the responsive subset of the network comprising 142 species (of which 53 are measured) and 195 reactions. We asked if the computational efficiency of our approach makes it applicable to construct large topologies. Results of this analysis are presented in more details in the “Supporting Information” section, S2 Text. We, finally, set out to compare the performance of our method with that of other predictive optimization methods, such as the ILP formulation presented in [42]. We focus on the differentiation of the two methods and we perform a fitness error calculation for the same input data. We, also, demonstrate a short statistical comparison through the Jaccard similarity index. For more information see S3 Text, in the “Supporting Information” section.

## Discussion

In this article, we introduce a novel approach, based on a graph algorithmic formulation, in order to construct cell-type specific pathways and to link signaling data to cellular responses. For the pathway construction, we take some generic networks of different scales and we combine them with experimental data. As a result, we present a new framework for interrogating and training signaling networks based on measurements from stimulus-signals experiments.

To do so, we investigated the possibility of shortest and alternative pathways finding, through the fundamental algorithms of graph theory. Applying this approach showed that through Warshall-Floyd algorithm and a Breadth First Search (BFS) traversal of the network, our method manages to construct a final topology, which satisfies as many experimental dependencies and achieves a significant fit of the data at hand. We defined the concept of network conflicts, as the inconsistencies between measurements and prior topologies [43]. Therefore, we sought an efficient computational formula, which differs from the optimization pipeline, to handle these mismatches. This computational formula concentrates on enumerating the satisfied dependencies and the existing conflicts in each “tested” pathway and accordingly gives a bonus to specific conflicts for their selection in the solution.

We presented our algorithmic procedure via a toy model demonstration, to make sure of the accuracy and the consistency of the solutions provided. Then, we combined data from realistic biological experiments with initial signaling topologies in order to eventually create networks that simulate the cell signaling process. We chose a medium scale and a large scale network, to evaluate the method’s features and to ascertain its ability to model any scale network. The products of our approach are computable models, which constitute a proof-of-principle that the proposed methodology can efficiently interrogate protein expression data.

To assess the computational sensitivity of our approach, we have also applied a cross-validation statistical analysis, in order to prove that our outputs are in conjuction only with the measurements and, thus, the probability of random solutions is excluded. We reached statistical distributions of expected forms describing in proper way the behavior and the incidence of the interrogated network’s reactions. Finally, to demonstrate the power of our approach, we proceeded to a comparison with the ILP method [42, 44]. Several similarities and differences can be noted between the two methods. Both approaches model signaling networks as interaction graphs and can be applied to network topologies stored in many databases, without the need to convert these graphs into other modeling formalisms. Additionally, they can both detect inconsistencies between measurements and network topologies. In contrast to our approach, ILP formulation determines an optimal subgraph of a given network that can reflect a scenario of measurements at best, using an optimization solver to guarantee the global optimal solution. This comparison, indicated the capacity of our algorithm to predict topologies similar to the state-of-the-art optimization pipelines.

To the best of our knowledge, the method introduced here, is the first that uses such an approach directly on interaction graphs to systematically interrogate and train the wiring diagrams of signaling networks. Since our methodology is strictly graph theory based, it displays limitations of static quality (while entities and relations between them, in biochemical networks, change with time, static network modeling can only capture a temporal aspect of experimental information flow [27, 45]). Another limitation is that our approach can handle only single treatment phosphoprotein activity and qualitative description of the experimental data. On the bright side, we sought to develop a fast computational technique to train the interrogated data, dropping significantly the fitness error and although we do not guarantee global optimum, using heuristic algorithms, we are able to identify the minimum supersets of the graph, where the optimum solution lies in. Avoiding the optimization algorithms that are typically NP-hard problems, we take advantage of the breadth-first transversal complexity of O(*V* + *E*) to impose the experimental dependencies. Furthermore, since this approach is only algorithmic, it does not require any optimization software (i.e. mathematical optimization-programming solvers) or environment to run and can be executed by any computer in any programming language implementation.

Signal transduction combines information regarding the activity state-concentration of the signaling components and the location of these components in the biochemical topologies. In the Introduction section, we referred to several modeling methodologies (e.g. Ordinary Differential Equations, Partial Differential Equations, etc.), which are valuable in describing the spatial dynamics of these signaling components and how the spatial signaling information is transduced from upstream to downstream components, within a known topology. However, when it comes to signal transduction, the role of the network topology is not easily deduced from such studies [30]. On the other hand, approaches like ours permit fast and qualitative network topology representation and predict cell-specific signaling pathways from stimulus-signals experiments. Therefore, our algorithm could assist end-users in the biological field and in pharmaceutical industry to understand and predict complex cellular regulations and to identify drug effects [46] by monitoring drug-induced topology alterations as described in [42]. As further research, it would also be interesting to pursue several extensions of our approach exploring potential combinations with optimization or probabilistic formulas, in order to achieve even better fit with the data at hand. Such combinations will present new mathematical and algorithmic challenges.

Overall, the approach described, successfully addresses the reconstruction of medium and large-scale signal transduction networks and provides a natural framework to enable the integrated analysis of proteomics. It allows the fast prediction of signaling topologies by combining the nature of graph theory with the flexibility of the pure programming, providing results comparable with the state-of-the-art optimization method ILP.

## Supporting Information

S1 Text Assessment of the model sensitivity–Cross-Validation analysis. (DOCX)

S2 Text Performance assessment in a large scale network. (DOCX)

S3 Text Comparison to ILP formulation. (DOCX)

S1 Fig Illustration of canonical and observable-controllable pathways. Observable-controllable part of the original topology. Numbers 57 species, 88 reactions and serves as starting point for the Cross-Validation analysis described in the “Results” section.
(EPS)

S2 Fig Medium scale network–Cross-Validation/Results presentation. We present the statistical analysis for the medium scale network. We ran the Cross-Validation analysis 500 times. In Y-axis we present the reactions incidence, while in X-axis we present the network reactions classified from the larger incidence to the smaller. Additionally, in blue we visualize the network reactions incidence after the 100% of the total runs, while in red we visualize the network reactions incidence after the 50% of the total runs. The main purpose is to demonstrate that our computational framework is sensitive to changes in experimental design (hence the random data generation), preserving the same generic topology and, thus, it does not favor the selection of specific network subsets. The inclusion of 50% and 100% cases, held to reach a convergence threshold prediction of these incidences.
(TIFF)

S3 Fig Medium scale network–Cross-Validation/Results visualization. We present the statistical analysis for the medium scale network. We ran the Cross-Validation analysis 500 times. In Y-axis we present the reactions incidence, while in X-axis we present the network reactions classified from the larger incidence to the smaller. Additionally, in blue we visualize the network reactions incidence after the 100% of the total runs, while in red we visualize the network reactions incidence after the 50% of the total runs. The main purpose is to demonstrate that our computational framework is sensitive to changes in experimental design (hence the random data generation), preserving the same generic topology and, thus, it does not favor the selection of specific network subsets. The inclusion of 50% and 100% cases, held to reach a convergence threshold prediction of these incidences.
(EPS)

S4 Fig Large scale network-Compressed model. The canonical pathway was constructed from literature. The experimental scenarios consist of 25 stimuli and 88 measured key phosphoproteins, as described properly in “The species translation challenge-A systems biology perspective on human and rat bronchial epithelial cells” [47]. Numbers 210 species, 473 reactions and this generic topology serves as a starting point for the analysis described in this paper. The model structure can be compressed substantially to 142 nodes and 195 edges. The compressed model reflects the essential dependencies in the original network structure, that can be addressed by the given set of the measured signals. Our solution resulted in a fitting error of 23, which has thus reduced much in comparison to 70 in original model. Our approach has successfully negotiated the construction of pathways to best fit the characteristics of the interrogated cell line. The pathway was built and visualized using Graphviz (http://www.graphviz.org/).
(EPS)
