# Supplementary material for: Network Reconstruction Based on Proteomic Data and Prior Knowledge of Protein Connectivity Using Graph Theory
Source: PLoS One. 2015 May 28;10(5):e0128411. doi: 10.1371/journal.pone.0128411 (PMC4447287; doi:10.1371/journal.pone.0128411)
Supplement: S1 Text — (DOCX) [file pone.0128411.s001.docx]

**S1 Text. Assessment of the model sensitivity - Cross-Validation analysis.**  Pathway pre-processing - Controllability and Observability: Inspecting the medium scale network (see "Medium scale network" subsection in the "Results" section) we observe that a large portion of the initial topology, is either non-perturbed (by the 5 stimuli) or non-observable. Thus it must be removed before the cross-validation analysis. Using Warshall's algorithm, we examine the connectivity of every node to the 16 signals and 5 stimuli. If we identify an existing pathway leading from at least a receptor to an arbitrary node A, then A is considered controllable. Additionally, if we identify another existing pathway from node A to at least one signal, then A is considered observable. The observable and controllable subset of the initial topology is plotted in S1 Fig.. S2 Fig. presents the statistical distribution of the reactions incidence after the total of 500 runs. Edges with 0% or 100% incidence are constant across all solutions, while edges with intermediate incidence vary from one solution to the other and thus, depend on the specific dataset. We observe that most edges are sensitive to the experimental data through not to the same degree. Some of them appear in more solutions than others, because of their important role in the satisfaction of specific dependencies. Overall, we observe that although our method produces solution of a specific structure, it is sensitive to variations in the experimental data, as it was expected. Finally, S3 Fig. presents the visualized results of this cross-validation analysis.
