# Supplementary material for: Network Reconstruction Based on Proteomic Data and Prior Knowledge of Protein Connectivity Using Graph Theory
Source: PLoS One. 2015 May 28;10(5):e0128411. doi: 10.1371/journal.pone.0128411 (PMC4447287; doi:10.1371/journal.pone.0128411)
Supplement: S2 Text — (DOCX) [file pone.0128411.s002.docx]

**S2 Text. Performance assessment in a large scale network.**  The construction procedure, as built around our formulation, receives as input the generic topology and the phosphoproteomic dataset, and by altering the topology that contradicts experimental measurements produces a compressed model that fits the experimental dataset with the least possible contradictions. The presented approach caused the fitness error to drop from 70% to 23%, validating that the initial topology alone was insufficient to properly describe the signal transduction mechanisms of the specific cell type. It, also, managed to fit the basic patterns of the experimental dataset shaping a descriptive model of how signal propagates in bronchial epithelial cells and preserved a subset of the reactions found in the PKN. Although manual inspection of the results is difficult, a few basic patterns can be identified. More specifically, 33 reactions of our solution are validated out of the set of 37 significant reactions published in [1]. The four reactions not validated by our solution are: CLENBUTEROL 🡪 ADR_FAMILY, GSK3B 🡪 TP53, MAPK3 🡪 TP53 and SRC 🡪 ABL1. Concluding, we presented an integrative approach to construct large scale signlaing pathways, based on phosphoproteomic data and a prior knowledge of protein connectivity. Our algorithmic formulation was able to prune an initial topology of 473 reactions and 210 nodes to satisfy the dependencies imposed by the dataset at hand and constitutes a proof-of-principle for construction of large-scale networks based on phosphoproteomic data.

[1]. Bilal,E., Sakellaropoulos,T., Participants,Challenge, Melas,I.N., Messinis,E.D., Rhrissorrakrai,K., et al., (2014) A crowd-sourcing approach for the construction of species-specific cell signaling networks, Bioinformatics, 10.1093/bioinformatics/btu659.
