# Supplementary material for: Network Reconstruction Based on Proteomic Data and Prior Knowledge of Protein Connectivity Using Graph Theory
Source: PLoS One. 2015 May 28;10(5):e0128411. doi: 10.1371/journal.pone.0128411 (PMC4447287; doi:10.1371/journal.pone.0128411)
Supplement: S3 Text — (DOCX) [file pone.0128411.s003.docx]

**S3 Text. Comparison to ILP formulation.**  The Jaccard index measures similarity between finite sample sets and is defined as the size of the intersection divided by the size of the union of the sample sets. Let A and B be two comparable sample sets, the Jaccard similarity index is defined as:

$$J\left( A,B \right)= \frac{\left| A\cap B \right|}{\left| A\cup B \right|}$$

Clearly, 0 ≤ J(A,B) ≤ 1.

In the case of the toy model we used to describe the logic process of our methodology, the Jaccard index demonstrated a 100% similarity between the solutions provided by the two methods.

As regards the medium scale network, the PKN demonstrated a fitness error of 59. Our method resulted in two solutions, according to the parameter change in our ranking method. The first one Fig.2 presented in the "Results" section) resulted in a final fitness error of 29 in a total time of 5 seconds, while the second one (Fig. 2 presented in the "Results" section - this new solution differs in the red edge) resulted in a final fitness error of 19, approximately in the same total time. On the other hand, the ILP method (maximum solution) resulted in a final fitness error of 6 in only 1 second. The Jaccard index between the two methods demonstrated for the first solution 65% similarity and for the second one 69% similarity.

Now as regards the large-scale network, the PKN demonstrated a fitness error of 70. Our method resulted, again, in two solutions, according to the parameter change in our ranking method. The first one resulted in a final fitness error of 24 in a total time of 76 seconds, while the second one (presented in S4 Fig.) resulted in a final fitness error of 23 in a total time of 56 seconds. On the other hand, the ILP method (maximum solution) resulted in a final fitness error of 7 in approximately two days running time. The Jaccard index between the two methods demonstrated for both solutions a 35% similarity.

Note that all calculations were done on a PC with a 2.13 GHz Intel double core Pentium P6200 CPU (only a single core was used) and 2 GB 1333 MHz DDR3 Memory.

The analysis above strongly indicated that the two methods produce quite similar results in the case of small scale and medium scale networks. In the first case the two methods tend to produce identical or quite similar solutions, whereas in the second one the similarity levels are quite sufficient. However, when it comes to larger networks, the two methods' solutions differ in the edges and the compounds selected for the compressed model. This differentiation depends on the fundamentals concepts underlying each methodology. More specifically, the method herein focus on the pathway construction based on shortest and alternative routes via a BFS searching algorithm, while the ILP optimization pipeline removes edges in order to reduce the objective function to global minimum. It is obvious that the remaining edges may not coincide with the shortest routes produced by Floy-Warshall algorithm. Hence, the similarity difference.
