# Supplementary figures and images for: Network Reconstruction Based on Proteomic Data and Prior Knowledge of Protein Connectivity Using Graph Theory
Source: PLoS One. 2015 May 28;10(5):e0128411. doi: 10.1371/journal.pone.0128411 (PMC4447287; doi:10.1371/journal.pone.0128411)

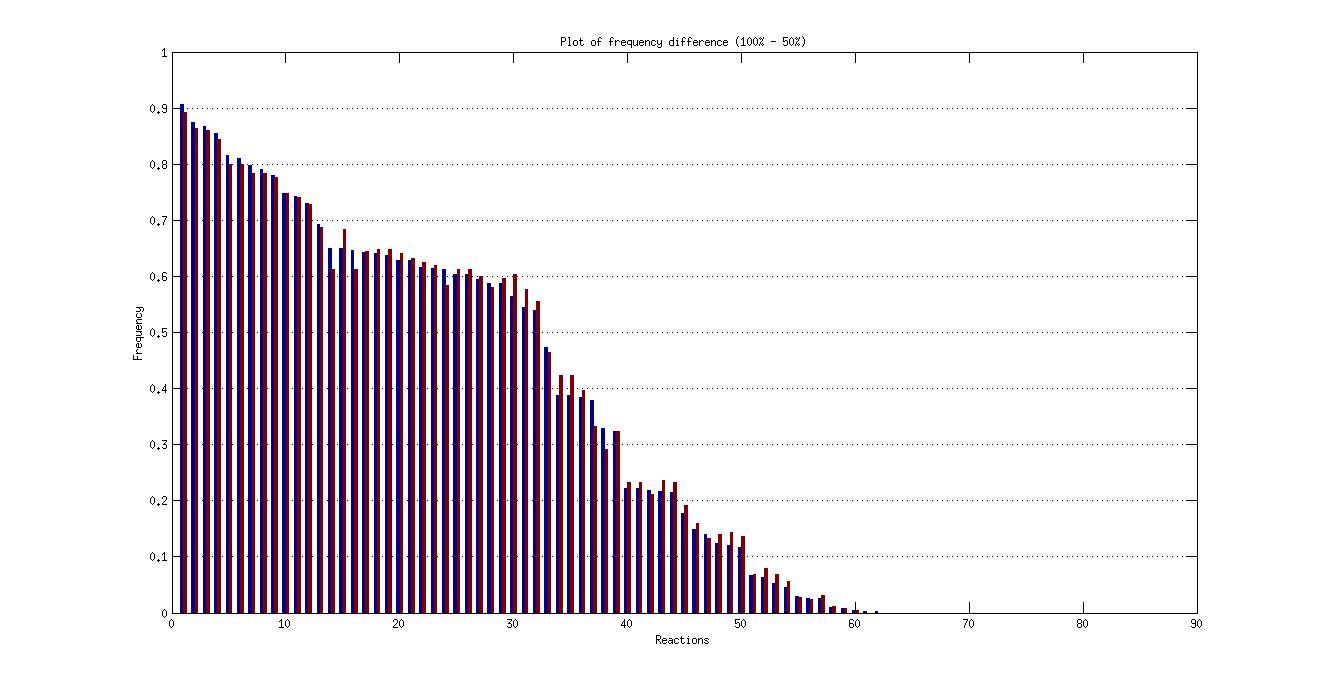

Supplement: S2 Fig — We present the statistical analysis for the medium scale network. We ran the Cross-Validation analysis 500 times. In Y-axis we present the reactions incidence, while in X-axis we present the network reactions classified from the larger incidence to the smaller. Additionally, in blue we visualize the network reactions incidence after the 100% of the total runs, while in red we visualize the network reactions incidence after the 50% of the total runs. The main purpose is to demonstrate that our computational framework is sensitive to changes in experimental design (hence the random data generation), preserving the same generic topology and, thus, it does not favor the selection of specific network subsets. The inclusion of 50% and 100% cases, held to reach a convergence threshold prediction of these incidences. (TIFF) [file pone.0128411.s005.tiff]
